# The survey of vaccination hesitancy among the residents in Jinan

**DOI:** 10.1371/journal.pone.0309431

**Published:** 2025-01-03

**Authors:** Dabing Wang, Shiyu Chen, Gaoyu Cui, Dequan Wang, Hong Liu, Lihui Zhao, Xiang Wang, Yuewei Chen

**Affiliations:** 1 The Department of Infectious Disease, General Hospital of Jinan Military Command: PLA 960th Hospital, Jinan, China; 2 Training Camp of The Shandong Armed Police Forces, Jinan, China; 3 The Department of Disease Control and Prevention, Shandong Armed Police Forces General Hospital, Jinan, China; 4 The Department of Oncology, Shandong Armed Police Forces General Hospital, Jinan, China; 5 The Department of Gynaecology and Obstetrics, Shandong Armed Police Forces General Hospital, Jinan, China; 6 The Second Department of Cadre Health, General Hospital of Jinan Military Command: PLA 960th Hospital, Jinan, China; Regional Health Care and Social Agency of Lodi, ITALY

## Abstract

**Introduction:**

Vaccination is an important way to prevent disease, but vaccine hesitancy will impact vaccine coverage and indirectly affect health. This study aims to survey the status of vaccine hesitancy among adults in Jinan.

**Methods:**

A cross-sectional study was conducted using the vaccine hesitancy scale among the parents of children and teenagers at hospitals in Jinan, China. We described the attitude of the parents to the vaccination through the dimensions of confidence (items: L1-L7) and the risk (items:L8-L10).The participants will be regarded as lacking confidence if the score is over 21 among the items (L1-L7), and participants will consider the vaccination to be a “Risk” if the score is over 9 among the items (L8-L10). Using the chi-square test to analyse the differences of attitude between different participants.

**Results:**

202 individuals were enrolled, and most respondents (88.70%) agreed that vaccines are important for their child’s health. 33.50% agreed and strongly agreed that new vaccines carried more risks than older vaccines. The average score for the lack of confidence in the vaccination was 11±0.25. The average score for risk for vaccination was 9.92±0.04. Participants aged below 30 years, females, those with lower education, and those without medical workers in the family were more concerned about the risks of vaccines.

**Conclusions:**

Participants were confident about the vaccination. But they were also concerned about the risks of vaccines. A lack of vaccine knowledge may led the participants to have hesitancy about vaccinations.

## Introduction

Vaccination was an effective method to improve health, especially for preventing infectious diseases. Many countries have made vaccination plans mandatory to improve the rate of vaccination [1, 2]. Vaccine hesitancy refers to a delay in acceptance or refusal of vaccination despite the availability of vaccination services [3], and vaccine hesitancy, as a considerable issue in European countries, has led to low coverage rates [4, 5]. Vaccine hesitancy refers not only to no-mandatory vaccines but also to mandatory vaccines. Vaccination hesitant has been reported for all the other vaccines in some European countries, including pneumococcal, measles and rubella [6]. Vaccination hesitancy will be more serious if getting the new infectious vaccine, such as the COVID-19 vaccine [7, 8]. That will increase the risk of contracting new infections and cause a heavy burden on society. Public confidence in vaccination loss has attracted great concern from multiple organizations (WHO, ECDC), and they warn of growing vaccine hesitancy and its impact on the declining trend of vaccine coverage [9, 10]. To find what influences vaccination hesitancy and increases vaccination coverage rates, many countries have done a lot of studies to find the factors, especially in high-income countries [11, 12]. The factors vary in different studies, including education, income, and demographic conditions [13, 14].

To improve the rate of vaccination in China, the government-funded Expanded Program on Immunization (EpI) and this program has had an obvious effect [15]. In China, many people are not actively vaccinated, especially for the new vaccine [16]. Shandong is one of the provinces in China. Jinan, as the provincial capital, has many residents and float population. If the float population cannot be vaccinated in time, the risk of infectious disease will increase and spread to other regions. Children are especially cared for by parents or other relatives and will be exposed to a more mobile population. This study aimed to describe the features of vaccine hesitancy among the parents of children and teenagers in Jinan and to compare vaccine hesitancy across different demographic groups.

## Methods

### Participants and procedures

We used the method of cross-sectional study to survey the adults. There are ten districts in Jinan, and the top five most densely populated districts were selected as survey sites. A convenience sampling method was used to recruit participants. The inclusion criterion was a parent or grandparent of a child <18years old of age. All participants had to be at least 18 years. Each participant was informed of the purpose of the study, and participants were assured that no one’s personal privacy would be disclosed. The sample size was calculated by the method of random sampling based on the vaccination rate of influenza vaccine. This study started from September 1, 2023 and ended on February 11 2024. We used the Vaccine Hesitancy Scale(VHS) which was developed by the WHO SAGE Working Group to address Vaccine Hesitancy [17] as the main questionnaire to survey the vaccination attitude. The other part of the questionnaire included information on socio-demographics, which included 10 items, and each item was assessed on a 5-score Likert scale. Fist seven items (L1-L7) were grouped into the component.

“Lack of confidence” and the other three items (L8-10) were grouped in to the component “Risk”. The options for each entry are assigned 5, 4, 3, 2, and 1 scores for the fist seven items. The options for each entry are assigned 1, 2, 3, 4, and 5 scores for the last three items. A higher score indicates a higher degree of vaccine hesitation. We define the participants as lacking confidence if the total score was over 21 among the items (L1-L7) and the outcomes were “risk” if the total score was over 9 among the items (L8-L10).The process of questionnaire survey was collected online and each participant was given publicity and guidance to ensure the authenticity of the data.

### Ethical statement

The study was conducted in accordance with the Declaration of Helsinki and approved by the Ethics Review Committee of Shandong Armed Police Forces General Hospital (Aug 25, 2023). And we obtained informed written consent. Participants gave written informed consent prior to data collection.

### Statistical analysis

Data analysis was performed using the SPSS Statistic 21.0. Frequency and percentage were applied to describe variables. Association analysis use the chi-square test. We analyzed the difference between different human attitude about the vaccine. *P* value < 0.05 was considered statistically significant. Using the logistic regression model to analyse which demographic characteristics influence the hesitation of residents to vaccinate. For the logistic regression models, the total score of the scale among the items(L8-L10) was treated as a dichotomous categoric variable accounting for either vaccination risk(score>9) or no risk(score≤9).

## Results

A total of 202 individuals were enrolled in the present study. Among the participants, The proportion of male was 25.90%. The average age was 36 years old. The proportion of families with one child was 56.80% and the proportion of participants with well educated (at least a bachelor’s degree) was 74.60%. The proportion of families with medical workers was 27.00%. The proportion of their children who were vaccinated for the first time was 71.40% (Table 1).

**Table 1:** Demographic variable and the association with vaccination risk.

vaccination risk(L8-L10) *p*-value				
Variable	n(%)	Risk (%)	no risk	
			(%)	
**Relation**				<0.01
Father	98(25.90)	45(45.90)	53(54.10)	
Mother	104(74.10)	70(67.30)	34(32.70)	
**Children gender**				0.17
boy	97(48.10)	50(51.50)	47(48.50)	
girl	105(51.90)	65(61.90)	40(38.10)	
**census register**				0.49
City	169(83.80)	98(58.00)	71(42.00)	
Village	33(16.20)	17(51.50)	16(48.50)	
**Educational attainment**				<0.01
Junior High school and lower	8(3.80)	5(62.50)	3(37.50)	
High school or junior college	44(21.60)	34(77.30)	10(22.70)	
Bachelor higher	150(74.60)	76(550.70)	74(49.30)	
**Age(years)**				0.02
≤30	26(13.00)	20(76.90)	6(23.10)	
>30	176(87.00)	95(54.00)	81(46.00)	
**Marital status**				
Single	0			
Married	202(100.00)	115(57.00)	87(43.00)	
**Number of children**				0.02
1	115(56.80)	75(65.20)	40(34.80)	
2	80(39.50)	37(46.30)	43(53.80)	
>2	7(3.70)	3(42.90)	4(57.10)	
**Employment status**				0.21
Employed	182(90.00)	101(55.50)	81(44.50)	
Unemployed	20(10.00)	14(70.00)	6(30.00)	
**Income**				0.37
<5000	40(20.00)	26(65.08)	14(34.92)	
5000–7500	57(28.10)	36(63.14)	21(36.86)	
>7500	105(51.90)	57(54.29)	48(45.71)	
**Whether to get vaccinated for the first time**				0.34
Yes	144(71.40)	79(54.90)	65(45.10)	
No	58(28.60)	36(62.10)	22(37.90)	
**Whether have a medical**				<0.01
**worker in family**				
Yes	55(27.00)	20(36.40)	35(63.60)	
No	147(73.00)	95(64.60)	52(35.40)

**Fig 1:**
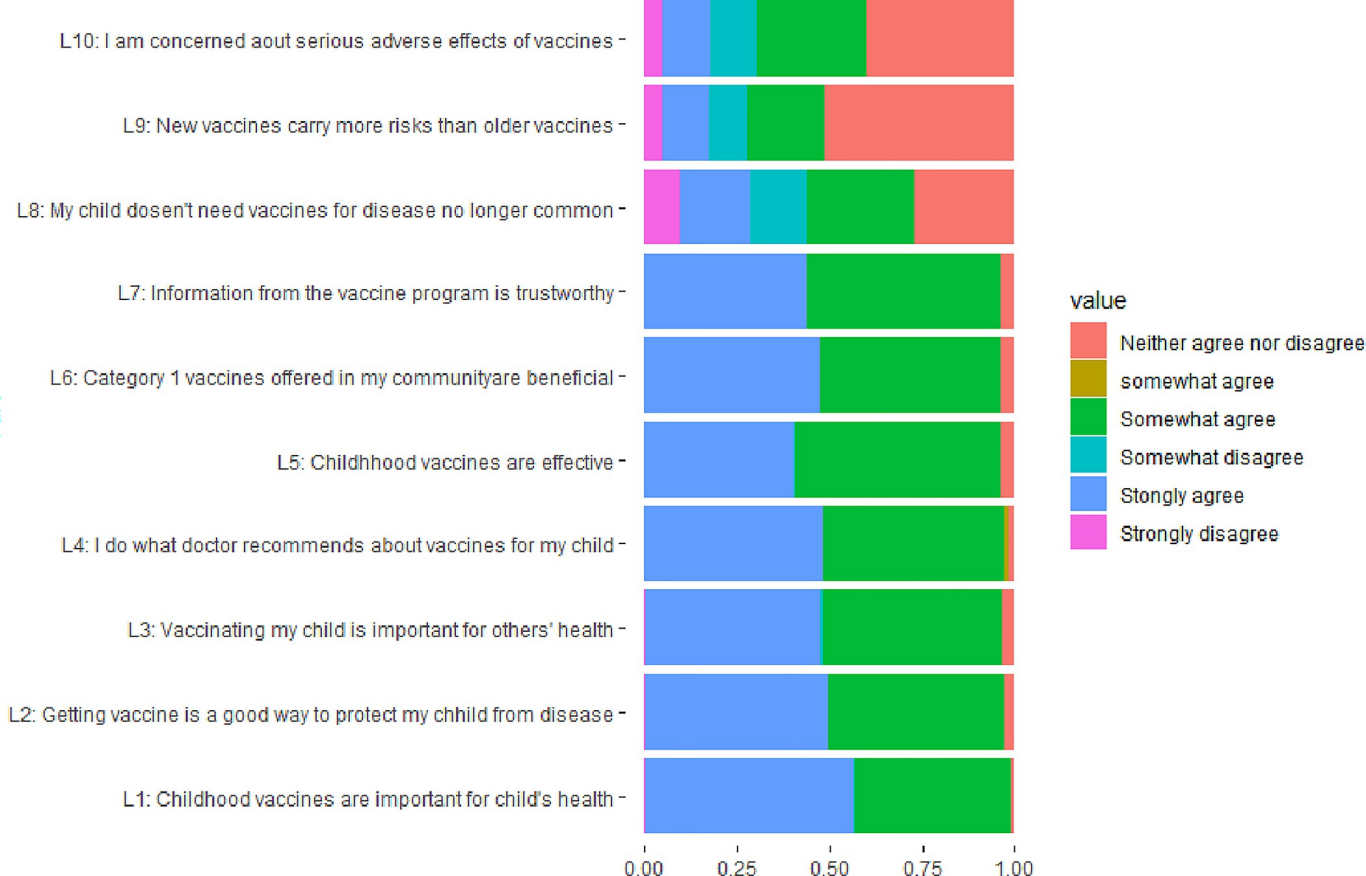
Distribution of responses to each item of the vaccine hesitancy scale.

1. Fig 1 shows the distribution of the responses for the VHS. The proportion that participants strongly agreed that vaccines are important for their child’s health was 55.70% and strongly agreed the vaccinating child is important for the healthy of others was 47.00%. The proportion that participants agreed and strongly agreed that new vaccines carried more risks than older vaccines was 33.50%.
2. The average score for the lack of confidence for the vaccination was 11, and no participant got total scores over 21. The average risk score for vaccination was 9.92±0.04. The proportion of participants who got total scores over 9 among the items(L8-L10) was 57.00%. Regarding risk, the female respondents had significantly higher scores (p <0.01) than males. The respondents in the young age group (< 30 years) and only with one child in the family had significantly high-risk scores (p  = 0.02). Respondents who had a higher level of education (Bachelor’s) had significantly lower risk scores (p<0.01). Without a medical worker, the family had significantly higher risk scores (p <0.01) (Table 1).

A logistic regression model was used to analyse the factors associated with the participants who were categorized as “at risk” (score>9) for the scale. The participants with age less than30 years (OR = 1.56), female (OR = 1.28), lower education(OR = 1.62), without medical worker in family (OR = 1.64) was more concerned about the risks of vaccines (Table 2).

**Table 2:** The factors influencing respondents with risk(score>9) attitude to the vaccination.

Factor		SE	*p*	OR(95%CI)
**Age**	<30	0.32	0.03	1.56(1.38–1.74)
**Gender**	Female	0.29	<0.01	1.28(1.23–1.53)
**The lever of Education**	Junior High school and lower	0.37	0.03	1.62(1.37–2.05)
**Whether have a medical worker in family**	No	0.53	0.02	1.64(1.58–1.83)

## Discussion

Jinan is the capital of Shandong, which has the most population, and the population mobility is significantly higher than that of other regions. So, the risk of infectious disease is much higher. Vaccination is the most effective way to prevent infectious disease. However, vaccination hesitancy will reduce vaccination rates, indirectly increasing the risk of infectious disease. In this study, we analysed the attitude toward vaccination. For the topic of “Lack of confidence”(L1-L7), most people were confident about the vaccination and thought it effectively protected their children. And people had a high awareness of the importance of vaccines. This implied that most participants trusted the vaccination supplied by government and had a high willingness to vaccinate their children. But there were fewer people with an ambiguous attitude to the items(L1-17). Those people were vaccine hesitators and may be likely to be potential non-vaccinators. If this group is migrant population, then they will be more likely to cause infectious disease. For the topic of “Risk” (L8-L10), the proportion of “Neither agree nor disagree” was higher than the proportion of other attitudes. And there was a higher proportion that “agree” the new vaccination was more risk than the “disagree”. Those two attitudes to the vaccination risk could reduce residents’ incentive to get new vaccines. With the change of climate and global mobility increasing. The new infections will become more common, and scientist will develop new vaccinations. If the rate of vaccination hesitancy is high, the new infections disease will be more difficult to control. Such as the new COVID-19 vaccine, lots of residents doubt the safety and refuse to get COVID-19 vaccinations [18–20].

Participants who are categorized as “risk” for the vaccination would be highly likely to refuse the vaccination. We analyzed the factors that may affect residents with a “risk” attitude toward vaccination. The lower age had a higher score than the older age. Most young parents have only one child in the family, and they may pay more attention to their children than those parents with more than one child. Young parents will have wider access to information and be more sensitive to some news, so they may be more likely to be influenced by negative news about vaccination [21]. People with lower education were more hesitant than those with higher education. People who with higher education possessed better knowledge about vaccination and correctly analyzed the advantages and disadvantages of vaccines. Some studies also found the level of hesitancy increased when the level of knowledge about the vaccine decreased, and people with better knowledge analysed the vaccine more thoroughly and responded positively [22, 23]. With a medical worker in the family, they may be more likely to give a lower score in the “Risk”. Medical worker is one of the strongest influencers in vaccination decisions, and they could provide the important and right information about the vaccines to the patients. In some European countries, medical workers were thought to be the most trustworthy sources of health alerts or information about medicines [24]. Doctors and nurses with high knowledge and understanding the effectiveness and safety of the vaccine were more likely to recommend vaccine to their relative or their friends [25, 26].

## Conclusions

In this study, we found most participants were confidence for the vaccination. But there also lots participants are concerned about the risks of vaccines which would become an important factor in vaccination hesitation. By disseminating the knowledge of vaccines may reduce the possibility of vaccine hesitancy. Health administrations also need to strengthen the promotion of vaccines and urging medical staff to enhance the explanation of vaccines, which will help to increase vaccination rates.
